# Effects of hyperbaric oxygen therapy on EEG and sEMG signals after low-load exercise

**DOI:** 10.7717/peerj.21554

**Published:** 2026-07-24

**Authors:** Beilun Fu, Miao Li, Qi Liu, Shufeng Zhou, Huan Zhu, Li Wan, Youling Qian

**Affiliations:** 1 Key Laboratory of Green Manufacturing of Super-Light Elastomer Materials of State Ethnic Affairs Commission, Hubei Minzu University, Ensen, China; 2 School of Sports, Hubei Minzu University, Enshi, China

**Keywords:** Blood flow restriction training, Acute exercise-induced fatigue, Hyperbaric oxygen therapy, Electroencephalogram signal, Surface electromyography signal, Blood flow restriction

## Abstract

**Objective:**

To evaluate the effect of hyperbaric oxygen therapy (HBOT) on acute exercise-induced fatigue via electroencephalogram (EEG) signal and surface electromyography (sEMG) signal, with the aim of providing a theoretical basis and methodological reference for promoting fatigue recovery. We hypothesized that HBOT for 60 min and natural recovery would reverse the changes in the indicators related to blood flow restriction (BFR) intervention, with HBOT demonstrating a superior restorative effect.

**Methods:**

College students (*n* = 34) majoring in physical education were randomly assigned to an experimental group (*n* = 17) or control group (*n* = 17). Fatigue was induced using four sets of weight-bearing squats (30 + 15 + 15 + 15 repetitions) combined with BFR intervention set to 80% of the arterial occlusion pressure (AOP) + 20% of the one repetition maximum (1RM) of the lower limbs. The control group naturally recovered for 60 min at ambient room temperature with 1.0 absolute atmospheres (ATA) and 20.9% oxygen concentration. The experimental group received 1.3 ATA hyperbaric oxygen therapy (HBOT) for 60 min. EEG signal, sEMG signal, heart rate, blood oxygen saturation, blood lactate accumulation (LA), and the Borg Subjective Fatigue Perception Assessment Scale (RPE) were assessed before exercise, immediately after exercise, and post-intervention.

**Results:**

LA, RPE, alpha and theta waves in the central area and occipital lobe; alpha waves in the parietal lobe; and average values of RMS, MF, and MPF of the musculus femoris medialis, musculus rectus femoris, and external sural muscle had time-group interaction effects. After intervention, the experimental group exhibited significantly higher α wave amplitudes (central, occipital, and parietal lobes) and MF and MPF values (vastus medialis, rectus femoris, and lateral gastrocnemius), compared with the control group (*P* < 0.05). By contrast, the experimental group demonstrated significantly lower β wave amplitudes in the central and occipital lobes, lower RMS values in the vastus medialis, rectus femoris, and lateral gastrocnemius muscles, and reduced LA levels and RPE scores, compared with the control group (*P* < 0.05).

**Conclusion:**

EEG and sEMG signal measurements indicate that BFR intervention induces central and peripheral fatigue simultaneously. After fatigue, 1.3 ATA-HBOT accelerates recovery from both types of fatigue within 60 min. Thus, this recovery method can be used as an effective strategy to accelerate recovery from BFR intervention-induced fatigue.

## Preface

Exercise fatigue is a temporary decline in exercise capacity and physical function caused by strenuous activity. Bejder et al. (2025) found that after high-intensity exercise at an intensity of 76% peak power, the massive accumulation of anaerobic glycolysis metabolites caused by tissue hypoxia is an important trigger for exercise-induced fatigue, and the degree of fatigue intensifies with increasing hypoxia. Hyperbaric oxygen therapy (HBOT) serves as an effective intervention to counter post-exercise hypoxia. HBOT involves inhaling high-concentration oxygen at pressures exceeding 1.0 atmosphere absolute (ATA). By increasing arterial oxygen partial pressure and circulatory oxygen reserves, HBOT accelerates oxygen transport and metabolic waste clearance, promoting recovery from exercise-induced fatigue (Qu et al., 2024). Owing to its non-invasive nature, portability, and lack of doping risk, HBOT has gained significant attention in sports science. Numerous studies have shown that administration of HBOT after High-load exercise can effectively promote the recovery from exercise-induced fatigue. Woo et al. (2020) conducted a 60-minute treadmill exercise at an intensity of 75–80% of maximum heart rate. They found that compared with the control group that only performed sedentary rest for the same duration, the experimental group receiving 60-minute HBOT intervention improved the body’s hypoxic state, accelerated the clearance of metabolic byproducts such as lactic acid, and alleviated oxidative stress response (Woo et al., 2020). Schottlender, Gottfried & Ashery (2021) also reached consistent conclusions in their review study, demonstrating that HBOT promotes recovery from high-load exercise-induced fatigue by alleviating hypoxia, enhancing the clearance of metabolic byproducts, and reducing oxidative stress. Its specific effects on exercise-induced fatigue have led to the widespread application of HBOT in sports training, where it has become a physical intervention for accelerating recovery from exercise-induced fatigue.

Exercise-induced fatigue is categorized into central and peripheral forms. Central fatigue is characterized by a progressive decline in voluntary activation or a reduction in neural stimulation to the muscles, ultimately decreasing maximum force generation capacity (Sadri, Khani & Sadri, 2014). Numerous studies have shown that characteristic changes in α waves and β waves can effectively reflect the functional state of the cerebral cortex, and their dynamic variations are closely related to the degree of fatigue, serving as electrophysiological indicators for evaluating exercise-induced changes in cerebral cortical function (Craig et al., 2012; Tanaka et al., 2012; Gutmann et al., 2018; Poinsard et al., 2025). Peripheral fatigue originates from functional changes at or distal to the neuromuscular junction, resulting in decreased maximum muscle force or power output (Wan et al., 2017). Numerous studies have shown that characteristic changes in surface electromyography (sEMG) amplitude, median frequency and mean power frequency can effectively reflect the functional status of skeletal muscle, and their dynamic variations are closely related to the degree of fatigue, serving as reliable electrophysiological indicators for evaluating exercise-induced changes in skeletal muscle function (Cifrek et al., 2009; Hsu et al., 2025; Leone et al., 2025).

However, most previous studies have adopted single-index detection. Measuring either signal in isolation fails to capture the fatigue state of the body, preventing an accurate assessment of fatigue development and a comprehensive evaluation of the effect of fatigue elimination. A fusion model using electroencephalogram (EEG) and sEMG time signals as node features achieved higher classification accuracy for exercise-induced fatigue than single-signal models (Yang, Li & Wang, 2022). EEG and sEMG data provide complementary information; thus, diagnosing exercise-induced fatigue *via* a single signal remains limited. However, their integration accurately reflects the functional state of the body (Yang, Li & Wang, 2022). In addition, EEG and sEMG testing are non-invasive, ensuring high compliance and acceptance among athletes. Thus, combining EEG and sEMG signals allows for a comprehensive assessment of both peripheral and central and an objective evaluation of recovery.

However, a review of the literature reveals no studies exploring the effect of HBOT on exercise-induced fatigue elimination *via* EEG and sEMG signals. Whether HBOT can simultaneously promote the recovery of EEG and sEMG signals remains inconclusive. Accordingly, this study evaluated the efficacy of HBOT in alleviating acute, exercise-induced fatigue through EEG and sEMG analysis, providing new evaluation method for the effect of HBOT on eliminating exercise-induced fatigue. We hypothesized that exercise-induced fatigue would significantly decrease α-wave amplitude and increase β-wave amplitude. In sEMG signals, we predicted an increase in RMS values alongside significant decreases in MF and MPF. Finally, we posited that while both HBOT for 60 min and natural recovery would reverse the changes in the aforementioned indicators, HBOT would demonstrate a superior restorative effect.

## Research Objects and Methodology

### Participants

In October 2024, participants were recruited through the official WeChat account of the Sports Science Center of Hubei University for Nationalities, campus posters, and campus on-site registration. The inclusion criteria were as follows: (1) male sports science majors who are competitive athletes with more than five years of professional training and experience in lower-limb resistance training (to reduce the potential confounding effects of hormonal fluctuations and menstrual cycles on test results); (2) no serious physical or psychiatric disorders; (3) no current use of medications or ongoing medical treatments; (4) no history of hypertension, coronary heart disease, rhinitis, respiratory tract infection, eustachian tube obstruction, otitis media, or dermatological conditions; (5) no claustrophobia; (6) no pulmonary dysfunction; (7) a willingness to participate, after being fully informed of the research objectives, procedures, test indices, and potential applications; and (8) clearance in accordance with the AHA/ACSM Health/Fitness Organization Pre-exercise Screening Questionnaire. After screening, the participants underwent electrocardiography, a cardiovascular function assessment, and a sports injury examination at the Affiliated Hospital of Hubei University for Nationalities (Grade A). The exclusion criteria were as follows: (1) presence of dermatological conditions, vascular or hematological blood diseases, cardiopulmonary conditions, or neurosensory disorders; (2) current severe sports injury or history of surgery within the past three years; (3) any relative or absolute contraindications to HBOT (*e.g.*, severe sinusitis, severe emphysema, pulmonary bullae, bronchiectasis.) (Silva, 2020); (4) contraindications to high-load exercise.

The a priori sample size estimation was conducted using G*Power 3.1 for a repeated-measures design incorporating intergroup–intragroup interactions. The minimum required sample size was 28 participants, as determined using an effect size *f* = 0.25, err prob α = 0.05, and power 1 − β = 0.80. The size was increased to consider potential loss due to sports injury and maladjustment induced by acute sports fatigue. Ultimately, 36 eligible participants were recruited and assigned *via* stratified block randomization. Body weight and skeletal muscle mass were estimated in kilograms (kg) using an Inbody 770 Body Composition Analyzer (South Korea) . Subjects were required to wear loose-fitting clothing for electrode contact and were prohibited from strenuous exercise or food intake within 2 h of testing. In addition, participants were instructed to remain calm to minimize muscle tension. First, participants were divided into subgroups (two participants per subgroup) based on comparable baseline skeletal muscle mass. Within each subgroup, participants were randomly assigned to either the natural relaxation (control) or HBOT (experimental) group, resulting in 18 participants per group. Detailed participant information is listed in Table 1. All participants were informed of the procedures and provided written informed consent. This study was approved by the Ethics Committee of Hubei MinZu University [2024 (072)] and the Chinese Clinical Trial Registry [ChiCTR2500109232].

### Experimental flow

**Figure 1:**
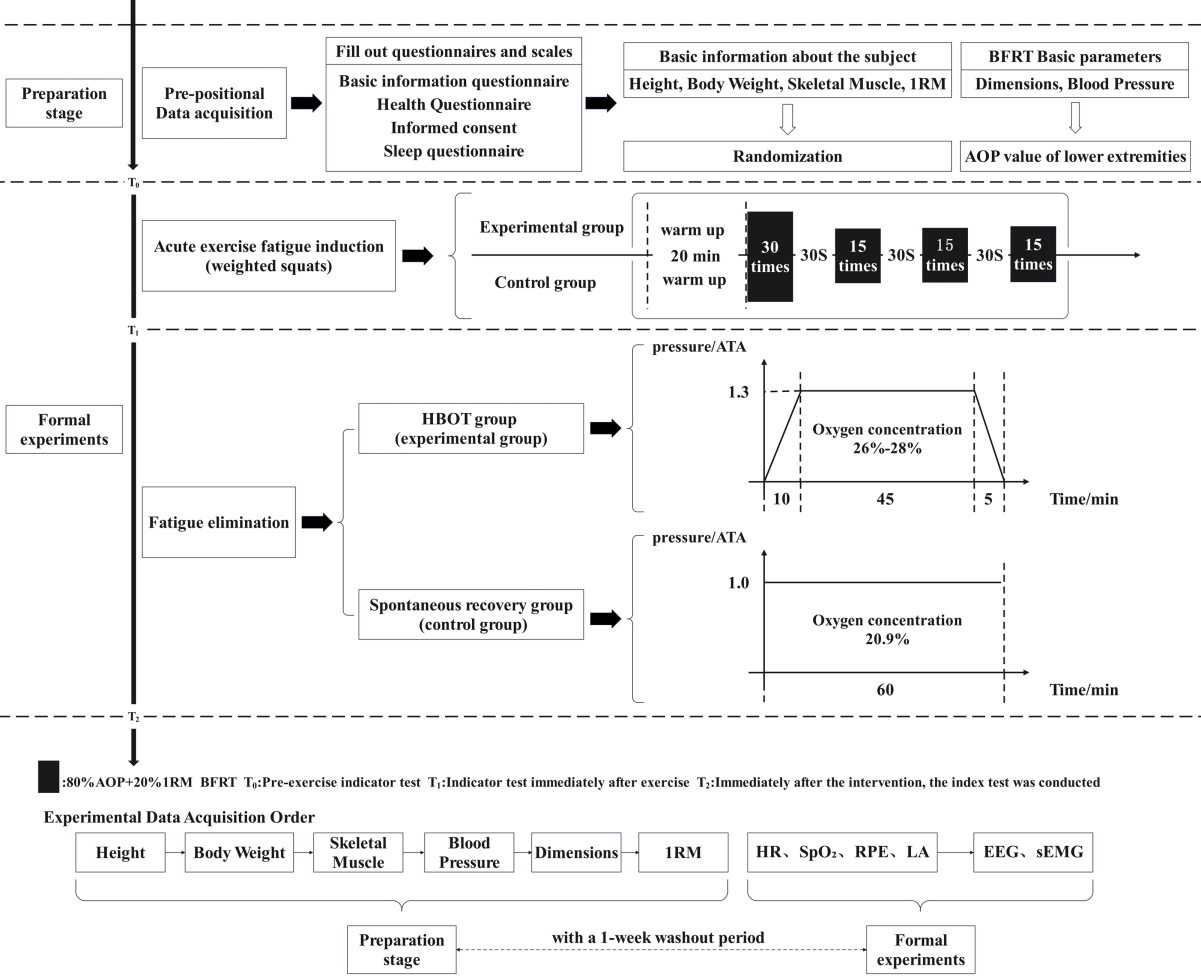
Experimental flow chart. Testing of various indicators, the time points for testing each indicator, and the entire experimental process (from top to bottom).

Baseline data were collected before the experiment: height, weight, one repetition maximum (1RM) value of lower limbs, dimension of lower limbs, and blood pressure. Arterial occlusion pressure (AOP) was calculated and, with the 1RM value of the lower limbs, was used to induce acute exercise fatigue. To determine 1RM, participants first completed a specific warm-up and then performed 10, 6, 3, and 1 repetitions in a non-fatigued state. Testers, blinded to group assignments, then conducted the 1RM squat test. During the test, a reasonable initial load was first selected based on the final sub-maximal lift. The load for each squat was then increased by a minimum of 5–15 kg per attempt until the participant could no longer maintain correct technique. The highest load successfully lifted was recorded as the 1RM (Bloomquist et al., 2013). After exercise, the experimental group received HBOT for 60 min, whereas the control group underwent natural recovery for 60 min (under normal pressure and oxygen). EEG signal, sEMG signal, blood oxygen saturation (SpO_2_), heart rate (HR), blood lactate accumulation (LA), and the Borg Subjective Fatigue Perception Assessment Scale (RPE) were measured before exercise, immediately after exercise, and immediately post-intervention. Figure 1 illustrates the specific experimental protocol.

**Table 1:** Demographic information of study participants.

Group	Number (name)	Age (years)	Height (cm)	Body weight (kg)	Training years (years)	Skeletal muscle (kg)
Experimental group	17	21.82 ± 1.63	177.02 ± 4.44	72.10 ± 4.69	6.06 ± 0.83	39.82 ± 3.64
Control group	17	21.00 ± 1.23	175.53 ± 3.72	74.05 ± 7.60	6.29 ± 0.77	37.64 ± 3.53
*t*		1.67	1.063	−0.902	−0.858	1.770
*P*		0.105	0.296	0.375	0.397	0.086

### Acute exercise fatigue induction program

Participants were instructed to refrain from strenuous exercise and the intake of stimulant beverages such as coffee within 12 h prior to the experiment (ensure adequate sleep), so as to ensure that they were in a good physical state before the test. Testing was conducted from 9:00 a.m. to 11:00 a.m. in the Exercise Physiology Laboratory of Hubei University for Nationalities (room temperature 20 °C–23 °C, humidity 45%–55%). Before the experiment, the participants were fitted with a Team Pro heart rate meter (Polar, Finland), an Aktos surface electromyography sensor (Cometa), and a NeuSen W EEG cap (Neuracle). Baseline data were recorded immediately before exercise.

During exercise, a 20-minute warm-up was conducted prior to the main activity. The session consisted of low-intensity aerobic exercise for 15 min to increase muscle temperature and circulation without inducing fatigue. Participants then performed two sets of three drop jumps with rest intervals for 1 min; these rapid eccentric–concentric contractions served to activate the musculature and induce post-activation potentiation enhancement (PAPE), enhancing subsequent force production. Concurrently, dynamic stretching and joint mobility exercises, such as dynamic lunges and hip rotations, were incorporated. Static stretching was strictly avoided to prevent the inhibition of explosive power. Finally, four 30-meter sprints were performed to recruit fast-twitch muscle fibers and optimize neuromuscular transmission, ensuring peak physiological readiness for the low-load protocol (McGowan et al., 2015).

Subsequently, acute fatigue was induced *via* a blood flow restriction (BFR) protocol using an intelligent pressure training instrument (KAATSU Master, Toyko, Japan). A compression belt five cm wide was bound to the proximal third of both thighs, positioned perpendicular to the longitudinal axis. The initial cuff pressure was set at 50 mmHg (Loenneke et al., 2015). AOP was determined based on thigh circumference, systolic blood pressure, and diastolic blood pressure (Table 2), as follows:

\begin{eqnarray*}{P}_{\mathrm{AOP}}=5.893l+0.734{P}_{\mathrm{DBP}}+0.912{P}_{\mathrm{SBP}}-220.046 \end{eqnarray*}

Note: where *P*_AOP_ is the occlusion pressure; *l* is the thigh circumference; *P*_DBP_ is diastolic pressure; and *P*_SBP_ is the systolic blood pressure.

**Table 2:** Basic parameters of blood flow restriction training in participants.

Group	Lower limb dimension (cm)		SBP (mmHg)	DBP (mmHg)	1RM (kg)	80% AOP	
	L	R				L	R
Experimental group	53.94 ± 6.79	54.73 ± 7.10	130.00 ± 12.03	76.06 ± 5.75	108.82 ± 13.41	217.77 ± 42.77	221.49 ± 44.14
Control group	54.51 ± 4.97	56.08 ± 5.50	128.76 ± 10.03	76.76 ± 9.22	106.71 ± 13.26	219.98 ± 35.07	227.35 ± 28.42

Participants completed weight-bearing squats under pressure, with BFR intervention intensity set at 80% AOP+20% of 1RM. The protocol consisted of four sets (30+15+15+15 repetitions), with an interval of 30s between sets (Fatela et al., 2016). At the start of training, the barbell was securely placed across the upper trapezius. Participants stood with feet positioned shoulder-width apart or slightly wider, toes slightly abducted, chest held out, abdomen engaged, core tightened, and spine in a neutral position. They squatted with hips and knees flexed and hips pushed backward. This position allowed the body to descend vertically until the thighs were parallel to the ground (or slightly lower). The knees were aligned with the toes to avoid inward buckling, and the heels remained in contact with the ground to stabilize the center of gravity. As the participants rose, they drove through their heels, pushing their buttocks and legs vertically while keeping the trunk stable without bending forward. The entire induction process was evaluated by two trained observers who assessed the execution in both the sagittal and coronal planes. During the study, two participants withdrew from the test due to pain in their lower limb joints during acute fatigue induction, failing to complete the experimental procedure.

### Recovery intervention

For the experimental group, a recovery intervention was administered using a portable soft oxygen chamber (model: OBI-I, China). Participants in this group entered the hyperbaric oxygen chamber, which was sealed and then pressurized, as described in the experimental protocol (Fig. 1). The chamber pressure was stabilized at 1.3 absolute atmospheres (ATA), with oxygen concentration at 26%–28%, temperature at about 22 °C ±  8 °C, and relative humidity at 40%–60%. Participants inhaled oxygen for 60 min under stable pressure to complete HBOT. After uniform pressure reduction for 5 min, participants exited the chamber. During the intervention, oxygen concentration was strictly monitored (Qu et al., 2024). One week before the formal experiment, participants underwent preconditioning for 5–10 min twice during the planned intervention period. They were informed about potential sensations, safety precautions, and emergency procedures. Their diet, work, and rest were strictly regulated to minimize errors caused by external factors. After an elution period of one week, formal experiments were conducted. Precautions: (1) In case of discomfort or an emergency, participants could adjust the pressure reduction valve inside the oxygen chamber or request assistance from a trained operator outside the chamber to reduce pressure and exit the chamber; (2) If participants experienced discomfort during the intervention, psychological counseling was provided so that the intervention would proceed, accompanied by trained operators throughout the process.

Meanwhile, participants in the control group sat quietly for 60 min under ambient room conditions (1.0 ATA, 20.9% oxygen concentration; 22 °C–28 °C). In order to minimize potential placebo effects, these participants were not informed about the research objectives of the study or educated on recovery methods. Neither group used electronics during recovery to eliminate the potential influence of electronic devices on test outcomes.

### Indicator test

Data were collected from both groups: before exercise, immediately after exercise, and immediately after recovery.

#### Primary outcome measures indicators

##### Electroencephalogram signal.

EEG signals were acquired using the NeuSen W (Neuracle, China) wireless acquisition system at 1,000 Hz sampling frequency. Two hours before testing, participants washed and dried their hair; 30 min before testing, they entered the Sports Physiology Laboratory of Hubei University for Nationalities and were fitted a with a 16-channel EEG cap. Electrode placement followed the international 10–20 system (Kim & Lee, 2018), with (reference electrode)REF as the reference electrode and (ground electrode) GND as the forehead electrode. GT-10 medical conductive paste produced by Greentec (China) was injected into all electrode holes, with the amount adjusted to maintain an electrode impedance level below five kΩ. EEG signals were collected with the participants’ eyes open at three time points: before exercise, after exercise, and after intervention. Signals corresponding to simulated testing and rest periods were removed.

The raw EEG signals were processed using MATLAB R2024a and its supporting EEGLAB program. Signals were filtered with a 0.5–60.0 Hz band-pass filter, followed by a 50 Hz notch filter. The filtered EEG data were segmented in 10 s intervals, and the EEG data were disassembled into typical independent components by independent component analysis. Interference artifacts, including blinks, ECG activity, power frequency interference, and head movement, were excluded.

The Welch method was used to calculate the power spectral density (PSD). Band power was determined using the formula ${f}_{ \left( x \right) }=\int \nolimits _{{f}_{1}}^{{f}_{2}}PSD(f)df$, for specific frequency ranges. The calculation process was completed using EEGLAB, and the result was expressed in µV^2^/Hz. Alpha waves (8–13 Hz) and beta waves (β-waves; 13–30 Hz) were selected as EEG evaluation indexes. The central (C3, C4), parietal (P3, P4), occipital (O1, O2), and temporal (T3, T4) areas were designated as the main observation areas.

##### Surface electromyography signal.

**Figure 2:**
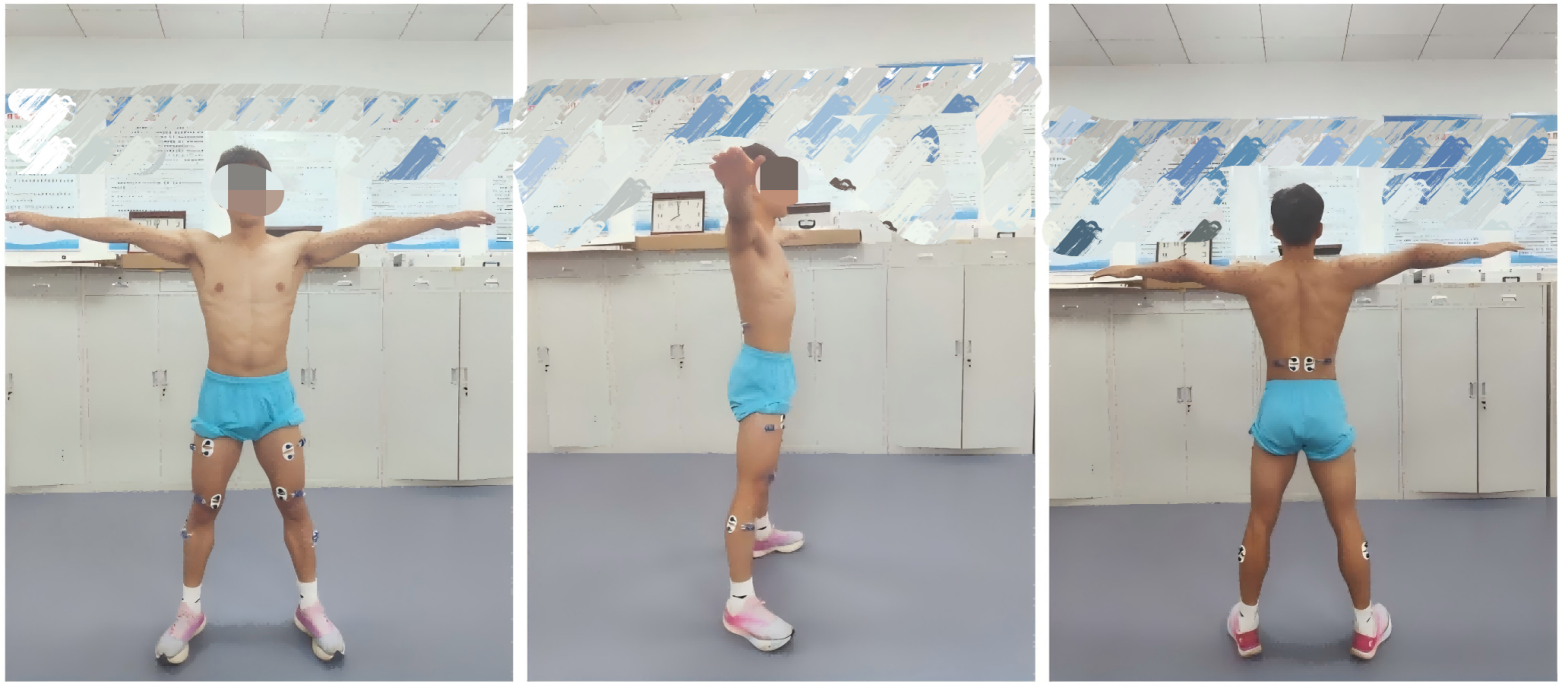
Surface electromyography signal position. This figure indicates the tested sEMG positions.

sEMG data were collected using the Aktos surface electromyography system, with electrodes placed on the following test sites: musculus femoris medialis, musculus rectus femor, erector spinae, and external sural muscle (Fig. 2). A Galaxy BookPro 360 laptop (Samsung, South Korea) and MX Brio camera (Logitech) were used to record sEMG activity during unloaded squats in subjects before exercise, immediately after exercise, and immediately after recovery, at a 1,000 Hz sampling rate. Surface electrodes were fixed at the test site with a 20 mm inter-electrode distance, and a reference electrode was positioned adjacent to it. Hair was shaved and skin cleaned skin before electrode placement. During preparation, participants stood upright, with feet shoulder-width apart, arms naturally relaxed at the sides, elbows slightly bent, palms relaxed, fingers slightly bent, and fingertips pointing downward. During testing, squats were performed with the hip joint as the axis: participants descended evenly and slowly, paused for 0.5–1 s at the lowest position, and rose quickly to return to the starting position.

After sEMG signal acquisition, data processing was conducted using the sEMG & Motion Tools program. An 8 Hz high-pass filter and a 500 Hz low-pass filter were applied to extract complete sEMG data from participants performing squats without weight loads (Izquierdo et al., 2011; Andersen et al., 2019). The average values of root mean square (RMS), median frequency (MF), and mean power frequency (MPF) in the frequency domain were selected as evaluation indices (Scano et al., 2020; Zhou, Li & Wang, 2011).

#### Secondary outcome measures indicators—routine monitoring indicators

To assess fatigue, this study used the Borg Rating of Perceived Exertion (RPE), heart rate (HR), blood oxygen saturation (SpO_2_), and LA (Pérez & Guedes, 2018; Zhao et al., 2024). RPE was measured using the standardized 6–20 Borg Scale. HR was monitored using a Team Pro heart rate monitor (Polar), and SpO_2_ was recorded from the distal phalanx of the right ring finger using a pulse oximeter (Wolman). Participants self-reported these scores simultaneously with the collection of other physiological metrics. LA concentration was measured from the distal phalanx of the right index finger using a nova portable blood lactate analyzer (SensLab GmbH). Before testing, trained professionals—blinded to the study groups—explained the Borg Scale (6–20) and clarified that scores must reflect a comprehensive subjective experience, including muscle fatigue, shortness of breath, and physical stress. To minimize bias, researchers remained blinded to group assignments throughout the study. The self-reported RPE scores of the participants were recorded simultaneously with other indicators (Scherr et al., 2013).

### Data processing

SPSS 27.0 and Microsoft Excel were used to complete the statistics and analysis of related data. Shapiro–Wilk was used to test the normal distribution of data; Levene’s method was used to test the homogeneity of variance of data; Mauchly sphericity test was used; and the Greenhouse-Geisser method was used to correct the sphericity hypothesis. Independent sample T test was used to compare the basic information of the two groups of subjects. A 3 (time: before exercise, after exercise, after intervention) ×2 (group: experimental group, control group) repeated measurement analysis of variance was used to compare the interaction between groups and time, and for the main effect analysis, simple effect analysis, and multiple comparison afterwards. The interaction effects were analyzed by Eta Squared. When the value of the interaction effect was less than 0.01, the effect was considered minimal; when the value was between 0.01 and 0.06, the effect was considered small; when the value was between 0.06 and 0.14, the effect was considered medium; and when the value 0.14 or higher, the effect was considered to be a large effect. The data results were expressed in the form of mean ±   standard deviation (M ±  SD), and the significance levels were *P* < 0.05.

## Research Results

### Comparison of changes in routine exercise fatigue indicators between two groups

**Figure 3:**
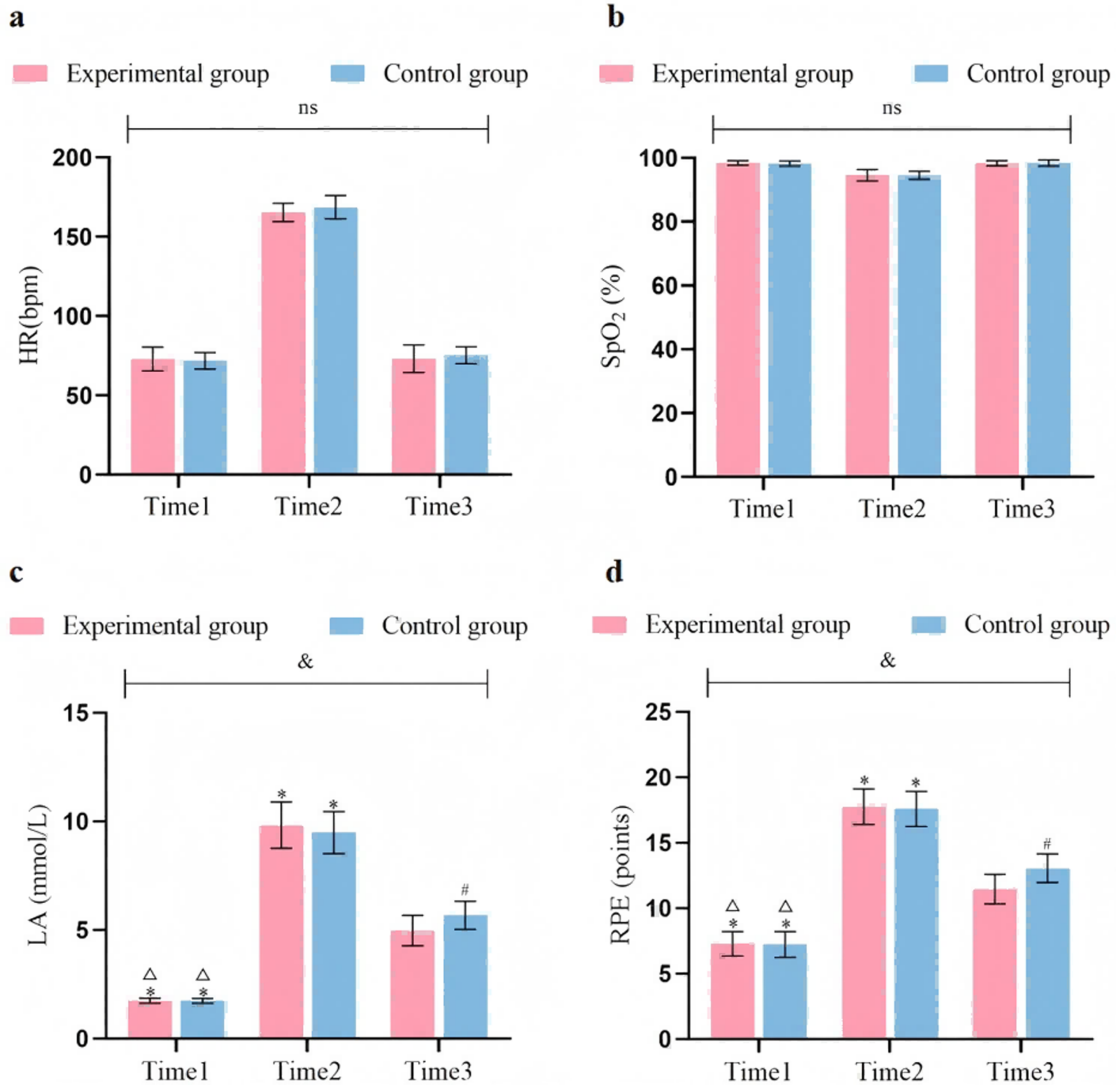
Comparison of the changes of routine monitoring indexes of exercise fatigue between the two groups. (A) Comparison of HR of participants in the two groups; (B) comparison of SpO2 of participants in the two groups; (C) comparison of LA of participants in the two groups; (D) comparison of RPE of participants in the two groups. Note: compared with the same group after intervention, ^∗^ *P* < 0.05; compared with the same group after exercise, ^Δ^*P* < 0.05; compared with the control group at the same time point, ^#^*P* < 0.05; the interaction between time and group, ^&^*P* < 0.05.

In the 3 (time: before exercise, after exercise, after intervention) ×2 (group: experimental group, control group) repeated measurement analysis of variance, there was an interaction between group and time for LA (*F* = 4.408, *P* = 0.012, *η*^2^_*p*_ = 0.121) and RPE (*F* = 7.260, *P* = 0.002, *η*^2^_*p*_ = 0.185; Table 3, Fig. 3). LA (*F* = 988.652, *P* < 0.05, *η*^2^_*p*_ = 0.969) and RPE (*F* = 808.912, *P* < 0.05, *η*^2^_*p*_ = 0.962) had significant main effects on time. The LA (*P* < 0.05, *η*^2^_*p*_ = 0.220) and RPE (*P* < 0.05, *η*^2^_*p*_ = 0.354) of participants in the control group was higher than that of participants in the experimental group; Simple effect was significant in the LA group (*P* <  0.05, *η*^2^_*p*_ = 0.979) and control group (*P* < 0.05, *η*^2^_*p*_ = 0.980), and significant in the RPE group (*P* < 0.05, *η*^2^_*p*_ = 0.954) and control group (*P* < 0.05, *η*^2^_*p*_ = 0.952). LA and RPE in the HBOT group were higher after exercise than before exercise (*P* < 0.05), after intervention (*P* < 0.05), and higher after intervention than before exercise (*P* < 0.05).

**Table 3:** Comparison of the changes in routine monitoring indexes of exercise fatigue between the two groups.

Fatigue index	Experimental group			Control group			*F*	*P*
	Before exercise	After exercise	After intervention	Before exercise	After exercise	After intervention		
HR (bpm)	72.88 ± 7.50	165.29 ± 5.73	73.06 ± 8.66	71.82 ± 5.16	168.59 ± 7.39	75.29 ± 5.34	1.010	0.369
SpO_2_ (%)	98.41 ± 0.71	94.59 ± 1.76	98.29 ± 0.85	98.24 ± 0.83	94.53 ± 1.23	98.38 ± 1.00	0.151	0.844
LA (mmol/L)	1.75 ± 0.12^*^^Δ^	9.83 ± 1.06^*^	4.98 ± 0.70	1.74 ± 0.11^*^^Δ^	9.49 ± 0.97^*^	5.68 ± 0.65^#^	4.408	0.016
RPE (points)	7.29 ± 0.92^*^^Δ^	17.76 ± 1.35^*^	11.47 ± 1.12	7.24 ± 0.97^*^^Δ^	17.59 ± 1.33^*^	13.06 ± 1.09^#^	7.260	0.002

**Notes.**

* Compared with the same group after intervention, *P* < 0.05.

** *P* < 0.01.

Δ Compared with the same group after exercise, *P* < 0.05.

ΔΔ *P* < 0.01.

# indicates that the group at the same time point is compared with *P* < 0.05.

## *P* < 0.01, The interaction between time and group.

& denotes *P* < 0.05.

&& denotes *P* < 0.01, the same below.

### Comparison of changes in EEG signal indexes between the two groups

#### Comparison of α-wave changes between the two groups

**Figure 4:**
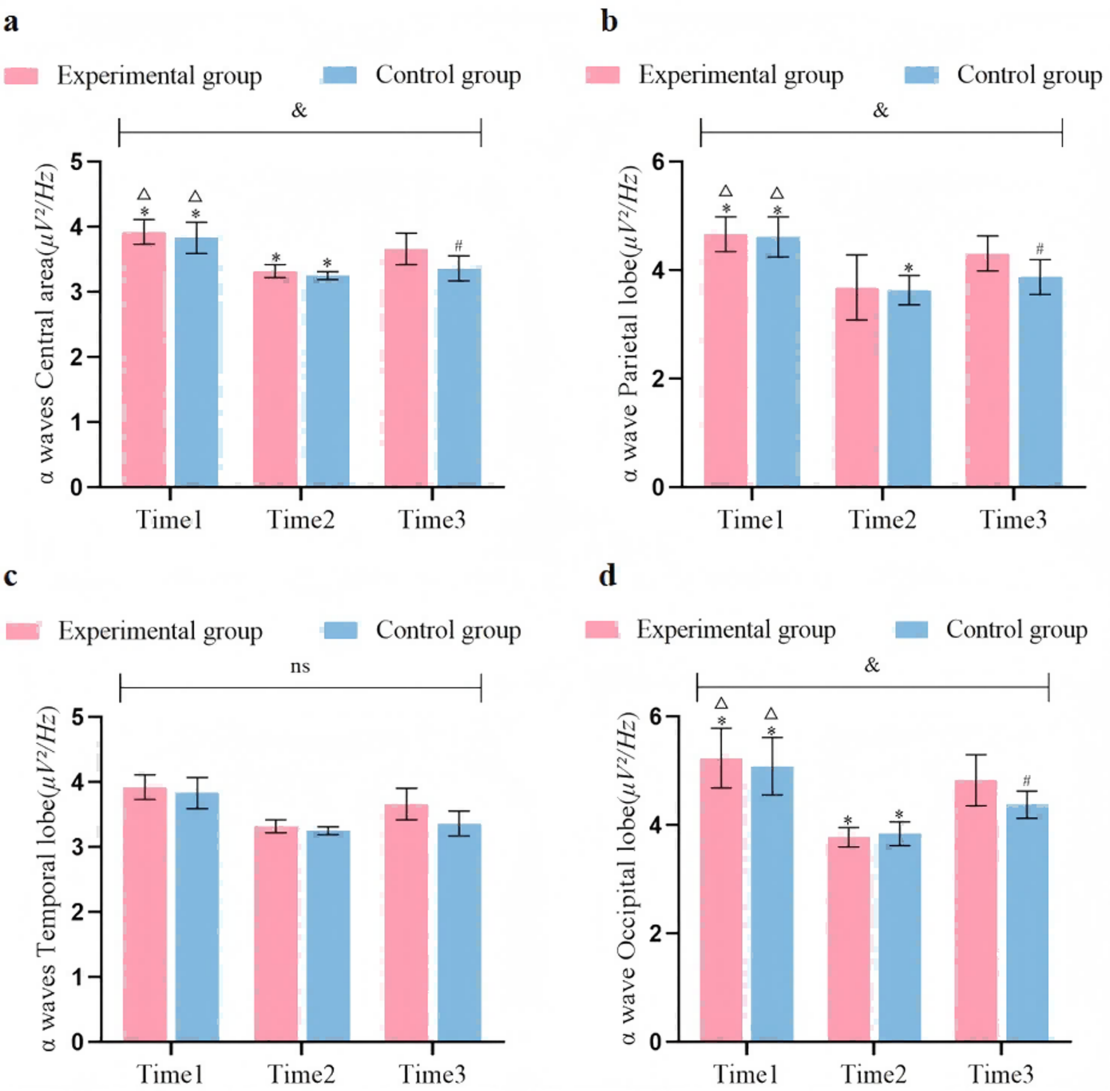
Comparison of EEG signal α-wave indexes between the two groups. (A) Comparison of α-waves in the central area of participants in the two groups; (B) comparison of α-waves in the p arietal lobe of participants in the two groups; (C) comparison of α-waves in the temporal lobe of participants in the two groups; (D) comparison of α-waves in the occipital lobe of participants in the two groups. Note: Compared with the same group after intervention, ^∗^ *P* < 0.05; compared with the same group after exercise, ^Δ^ *P* < 0.05; compared with the control group at the same time point, ^#^ *P* < 0.05; the interaction between time and group, ^&^ *P* < 0.05.

There was an interaction between group and time in the α-wave changes of the central area (*F* = 4.657, *P* = 0.013, *η*^2^_*p*_ = 0.127), parietal lobe (*F* = 3.525, *P* = 0.042, *η*^2^_*p*_ = 0.099), and occipital lobe (*F* = 5.965, *P* = 0.004, *η*^2^_*p*_ = 0.157; Table 4, Fig. 4). The main effects of time on α-wave changes in the central area (*F* = 97.082, *P* < 0.05, *η*^2^_*p*_ = 0.752), parietal lobe (*F* = 65.530, *P* < 0.05, *η*^2^_*p*_ = 0.672), and occipital lobe (*F* = 159.284, *P* < 0.05, *η*^2^_*p*_ = 0.833) were significant. The α-wave changes in the central area (*P* < 0.05, *η*^2^_*p*_ = 0.338), parietal lobe (*P* < 0.05, *η*^2^_*p*_ = 0.340), and occipital lobe (*P* <  0.05, *η*^2^_*p*_ = 0.276) of participants in the control group were lower than those of participants in the experimental group. The α-wave differences reached significance in the central area in both the experimental group (*P* < 0.05, *η*^2^_*p*_ = 0.842) and the control group (*P* < 0.05, *η*^2^_*p*_ = 0.772), in the parietal lobe in both the experimental group (*P* < 0.05, *η*^2^_*p*_ = 0.693) and the control group (*P* <  0.05, *η*^2^_*p*_ = 0.765), and in the occipital lobe in both the experimental group (*P* < 0.05, *η*^2^_*p*_ = 0.890) and the control group (*P* <  0.05, *η*^2^_*p*_ = 0.802). In the experimental group, the α-wave values of the central area, the parietal lobe, and the occipital lobe after exercise were lower than those before exercise (*P* < 0.05) and after intervention (*P* < 0.05), and the values after intervention were lower than those before exercise (*P* < 0.05). In the control group, the α-wave values of the parietal lobe after exercise were lower than those before exercise (*P* < 0.05), but not significantly different from those after intervention (*P* >  0.05), while the values after intervention were lower than those before exercise (*P* < 0.05).

**Table 4:** Comparison of EEG Signal α-wave indexes between the two groups (µV^2^/Hz).

Fatigue index	Experimental group			Control group			*F*	*P*
	Before exercise	After exercise	After intervention	Before exercise	After exercise	After intervention		
Central area	3.92 ± 0.19^*^^Δ^	3.32 ± 0.10^*^	3.66 ± 0.24	3.83 ± 0.24^*^^Δ^	3.25 ± 0.06^*^	3.36 ± 0.19^#^	4.657	0.013
Parietal lobe	4.66 ± 0.32^*^^Δ^	3.68 ± 0.60	4.31 ± 0.32	4.61 ± 0.37^*^^Δ^	3.63 ± 0.27^*^	3.87 ± 0.32^#^	3.525	0.042
Temporal lobe	2.90 ± 0.31	2.35 ± 0.26	2.61 ± 0.35	2.98 ± 0.21	2.37 ± 0.26	2.58 ± 0.28	0.328	0.709
Occipital lobe	5.23 ± 0.55^*^^Δ^	3.77 ± 0.18^*^	4.82 ± 0.47	5.08 ± 0.53^*^^Δ^	3.84 ± 0.22^*^	4.37 ± 0.25^#^	5.965	0.004

**Notes.**

Compared with the same group after intervention.

* *P* < 0.05; Compared with the same group after exercise.

Δ *P* < 0.05; Compared with the control group at the same time point.

# *P* < 0.05; The interaction between time and group.

& *P* < 0.05.

#### Comparison of β -wave changes between groups

**Figure 5:**
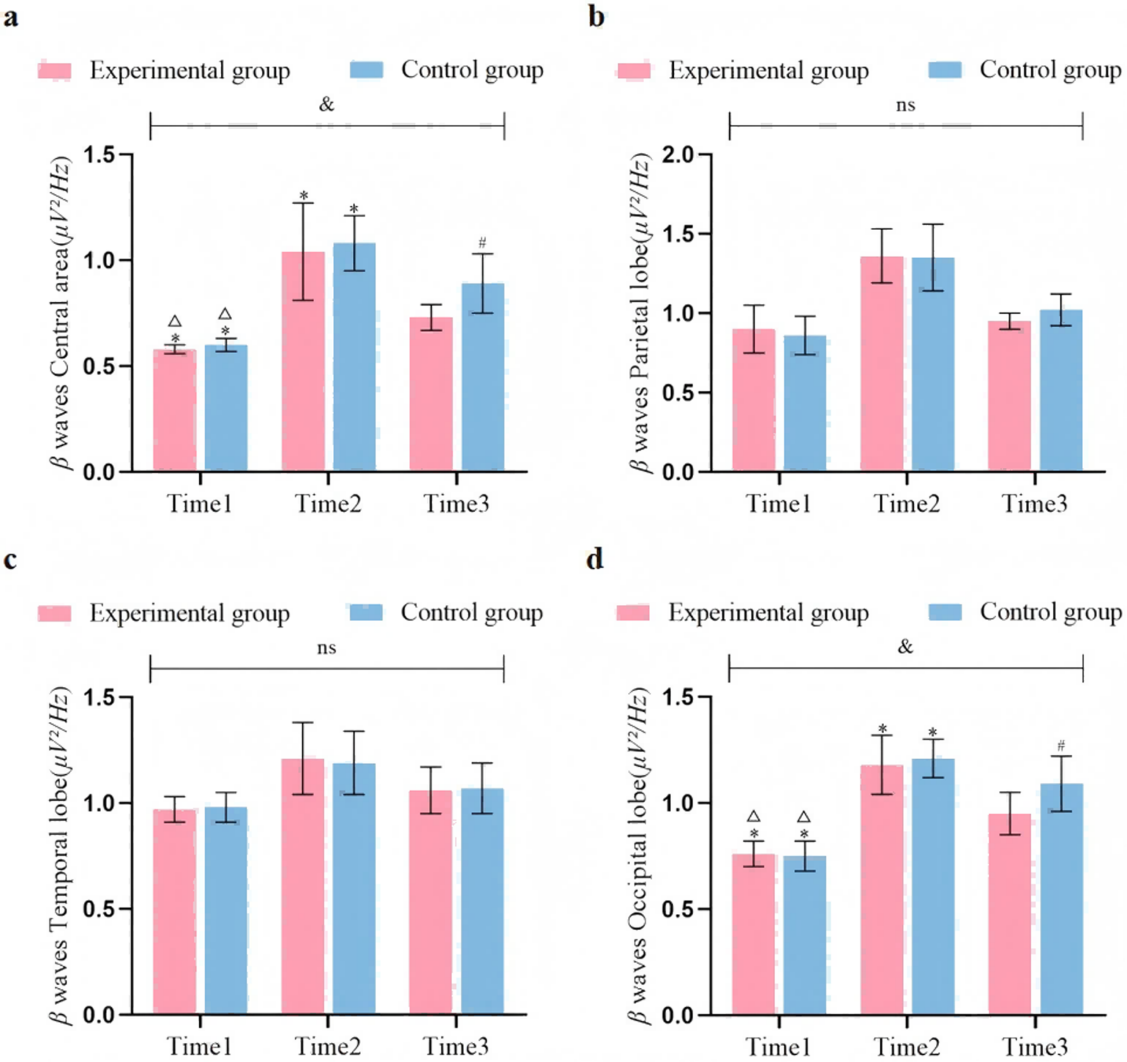
Comparison of EEG signal β-Wave indexes between the two groups. (A) Comparison of β-waves in the central area of participants in the two groups; (B) comparison of β-waves in the parietal lobe of participants in the two groups; (C) comparison of β-waves in the temporal lobe of participants in the two groups; (D) comparison of β-waves in the occipital lobe of participants in the two groups. Note: Compared with the same group after intervention, ^∗^ *P* < 0.05; Compared with the same group after exercise, ^Δ^ *P* < 0.05; compared with the control group at the same time point, ^#^ *P* < 0.05; the interaction between time and group, ^&^ *P* < 0.05.

There was an interaction between group and time in the β -wave changes of the **c**entral area (*F* = 4.667, *P* = 0.013, *η*^2^_*p*_ = 0.127) and occipital lobe (*F* = 5.102, *P* = 0.011, *η*^2^_*p*_ = 0.138); Table 5, Fig. 5). The main effects of time on β -wave changes in the central area (*F* = 170.165, *P* < 0.05, *η*^2^_*p*_ = 0.842) and occipital lobe (*F* = 153.198, *P* < 0.05, *η*^2^_*p*_ = 0.827) were significant. The β -wave changes in the central area (*P* < 0.05, *η*^2^_*p*_ = 0.375) and occipital lobe (*P* < 0.05, *η*^2^_*p*_ = 0.285) of participants in the control group were higher than those of participants in the experimental group. The β -wave changes reached significance in the central area of both the experimental group (*P* < 0.05, *η*^2^_*p*_ = 0.778) and the control group (*P* < 0.05, *η*^2^_*p*_ = 0.855) and in the occipital lobe of both the experimental group (*P* <  0.05, *η*^2^_*p*_ = 0.845) and the control group (*P* < 0.05, *η*^2^_*p*_ = 0.891). The β-wave changes in the central area and occipital lobe in the same group were higher after exercise than before exercise (*P* < 0.05), higher after exercise than after intervention (*P* < 0.05), and higher after intervention than before exercise (*P* < 0.05).

### Comparison of sEMG signal changes between groups

#### Comparison of RMS changes between groups

**Figure 6:**
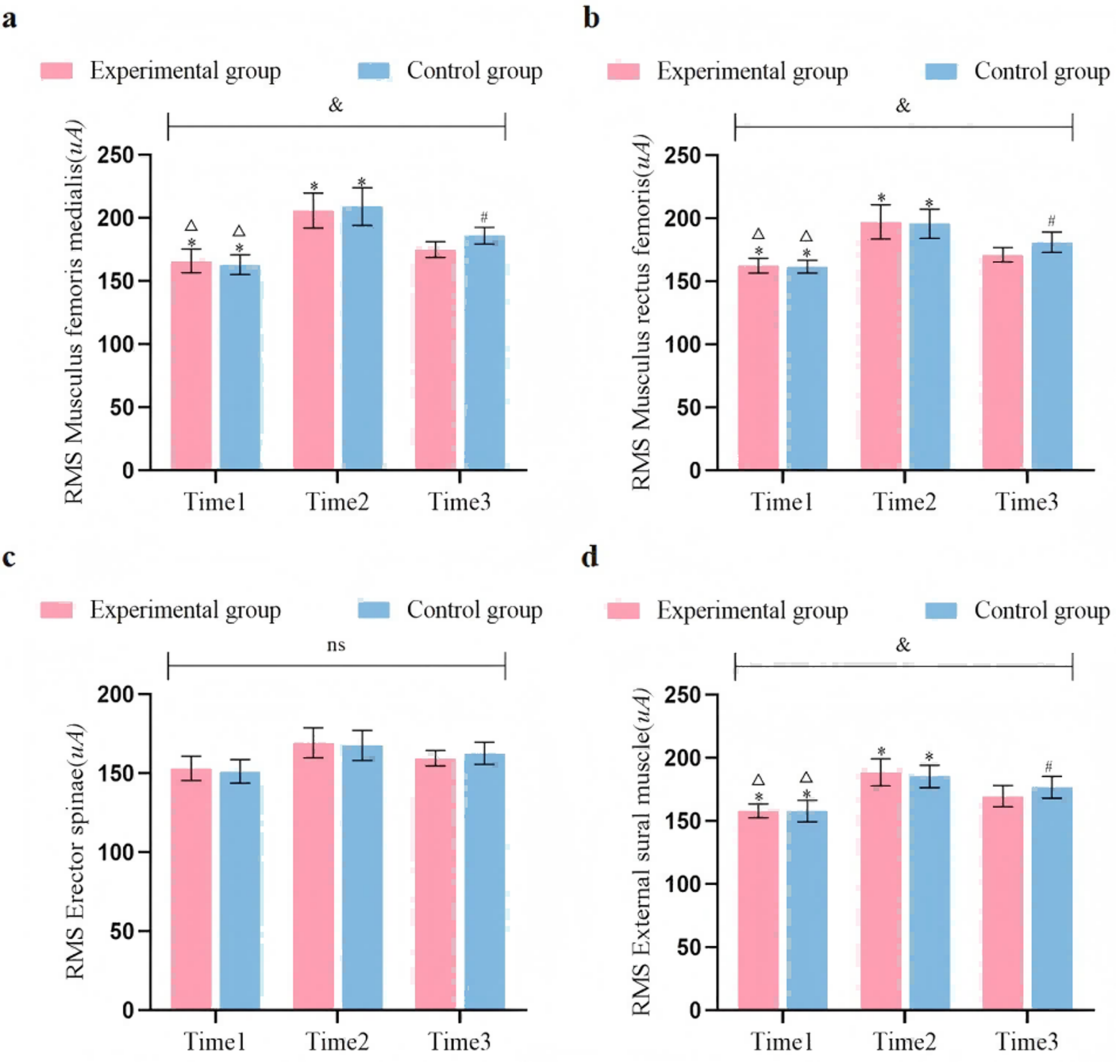
Comparison of sEMG signal RMS indexes between the two groups. (A) Comparison of RMS in the m edial femoral muscle of participants in the two groups; (B) comparison of RMS in the musculus rectus femoris of participants in the two groups; (C) comparison of RMS in the erector spinae of participants in the two groups; (D) comparison of RMS in the external sural muscle of participants in the two groups. Note: Compared with the same group after intervention, ^∗^ *P* < 0.05; compared with the same group after exercise, ^Δ^ *P* < 0.05; compared with the control group at the same time point, ^#^ *P* < 0.05; the interaction between time and group, ^&^ *P* < 0.05.

There was an interaction between group and time in the RMS of the musculus femoris medialis (*F* = 4.859, *P* = 0.011, *η*^2^_*p*_ = 0.132), musculus rectus femoris (*F* = 7.503, *P* = 0.001, *η*^2^_*p*_ = 0.190), and external sural muscle (*F* = 6.141, *P* = 0.006, *η*^2^_*p*_ = 0.161; Table 6, Fig. 6). The main effects of time on RMS changes in the musculus femoris medialis (*F* = 187.244, *P* < 0.05, *η*^2^_*p*_ = 0.854), musculus rectus femoris (*F* = 215.422, *P* < 0.05, *η*^2^_*p*_ = 0.871) and external sural muscle (*F* = 181.944, *P* < 0.05, *η*^2^_*p*_ = 0.850) were significant. After intergroup intervention, the RMS in the musculus femoris medialis (*P* < 0.05, *η*^2^_*p*_ = 0.442), rectus femoral muscle (*P* <  0.05, *η*^2^_*p*_ = 0.353), and external sural muscle (*P* < 0.05, *η*^2^_*p*_ = 0.153) of the participants in the control group were higher than those of the participants in the experimental group. RMS changes reached significance in the musculus femoris medialis of both the experimental group (*P* < 0.05, *η*^2^_*p*_ = 0.814) and the control group (*P* <  0.05, *η*^2^_*p*_ = 0.874), in the rectus femoral muscle of both the experimental group (*P* < 0.05, *η*^2^_*p*_ = 0.849) and the control group (*P* <  0.05, *η*^2^_*p*_ = 0.911), and in the external sural muscle group of both the experimental group (*P* < 0.05, *η*^2^_*p*_ = 0.910) and the control group (*P* < 0.05, *η*^2^_*p*_ = 0.896). In the musculus femoris medialis, musculus rectus femoris, and external sural muscle, RMS values within the same group were higher after exercise than before exercise (*P* < 0.05) and after intervention (*P* < 0.05), with values after intervention also being higher than those before exercise (*P* < 0.05).

**Table 5:** Comparison of EEG signal β-wave indexes between the two groups (µV^2^/Hz).

Fatigue index	Experimental group			Control group			*F*	*P*
	Before exercise	After exercise	After intervention	Before exercise	After exercise	After intervention		
Central area	0.58 ± 0.02^*^^Δ^	1.04 ± 0.23^*^	0.73 ± 0.06	0.60 ± 0.03^*^^Δ^	1.08 ± 0.13^*^	0.89 ± 0.14^#^	4.667	0.013
Parietal lobe	0.90 ± 0.15	1.36 ± 0.17	0.95 ± 0.05	0.86 ± 0.12	1.35 ± 0.21	1.02 ± 0.10	2.158	0.124
Temporal lobe	0.97 ± 0.06	1.21 ± 0.17	1.06 ± 0.11	0.98 ± 0.07	1.19 ± 0.15	1.07 ± 0.12	0.281	0.756
Occipital lobe	0.76 ± 0.06^*^^Δ^	1.18 ± 0.14^*^	0.95 ± 0.10	0.75 ± 0.07^*^^Δ^	1.21 ± 0.09^*^	1.09 ± 0.13^#^	5.102	0.011

**Notes.**

Compared with the same group after intervention.

* *P* < 0.05; Compared with the same group after exercise.

Δ *P* < 0.05; Compared with the control group at the same time point.

# *P* < 0.05; The interaction between time and group.

& *P* < 0.05.

#### Comparison of MF changes between the two groups

**Figure 7:**
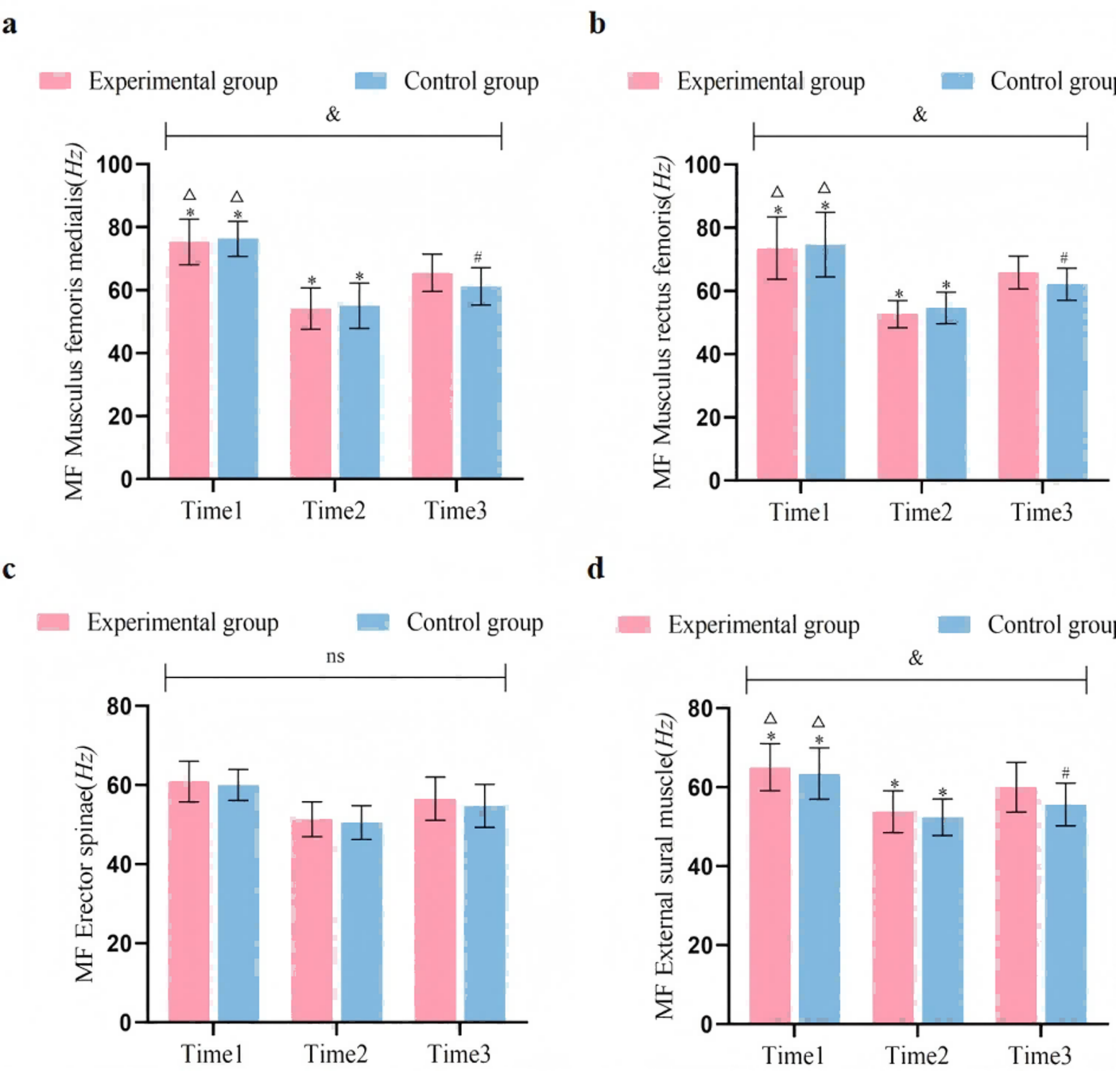
Comparison of changes in sEMG signal MF indexes between the two groups. (A) Comparison of MF in the medial femoral muscle of participants in the two groups; (B) comparison of MF in the musculus rectus femoris of participants in the two groups; (C) comparison of MF in the erector spinae of participants in the two groups; (D) comparison of MF in the external sural muscle of participants in the two groups. Note: Compared with the same group after intervention, ^∗^ *P* < 0.05; compared with the same group after exercise, ^Δ^*P* < 0.05; compared with the control group at the same time point, ^#^ *P* < 0.05; the interaction between time and group, ^&^ *P* < 0.05.

There was an interaction between group and time in the MF of the musculus femoris medialis (*F* = 3.479, *P* = 0.037, *η*^2^_*p*_ = 0.098), musculus rectus femoris (*F* = 3.749, *P* = 0.029, *η*^2^_*p*_ = 0.105), and external sural muscle (*F* = 3.343, *P* = 0.045, *η*^2^_*p*_ = 0.095; Table 7, Fig. 7). The main effects of time on MF changes in the musculus femoris medialis (*F* = 168.587, *P* < 0.05, *η*^2^_*p*_ = 0.840), musculus rectus femoris (*F* = 171.275, *P* < 0.05, *η*^2^_*p*_ = 0.843), and external sural muscle (*F* = 153.445, *P* < 0.05, *η*^2^_*p*_ = 0.827) were significant. The MF of the musculus femoris medialis (*P* < 0.05, *η*^2^_*p*_ = 0.124), rectus femoral muscle (*P* < 0.05, *η*^2^_*p*_ = 0.121), and external sural muscle (*P* < 0.05, *η*^2^_*p*_ = 0.128) of participants in the control group were lower than those of participants in the experimental group. MR changes reached significance in the musculus femoris medialis in both the experimental group (*P* < 0.05, *η*^2^_*p*_ = 0.814) and the control group (*P* <  0.05, *η*^2^_*p*_ = 0.869), in the rectus femoral muscle group of both the experimental group (*P* < 0.05, *η*^2^_*p*_ = 0.888) and the control group (*P* < 0.05, *η*^2^_*p*_ = 0.804), and in the external sural muscle of both the experimental group (*P* < 0.05, *η*^2^_*p*_ = 0.823) and the control group (*P* < 0.05, *η*^2^_*p*_ = 0.805). In the musculus femoris medialis, musculus rectus femoris, and external sural muscle, the MF values within the same group were lower after exercise than before exercise (*P* < 0.05) and after intervention (*P* < 0.05), with values after intervention also being lower than those before exercise (*P* < 0.05).

#### Comparison of MPF changes between groups

**Figure 8:**
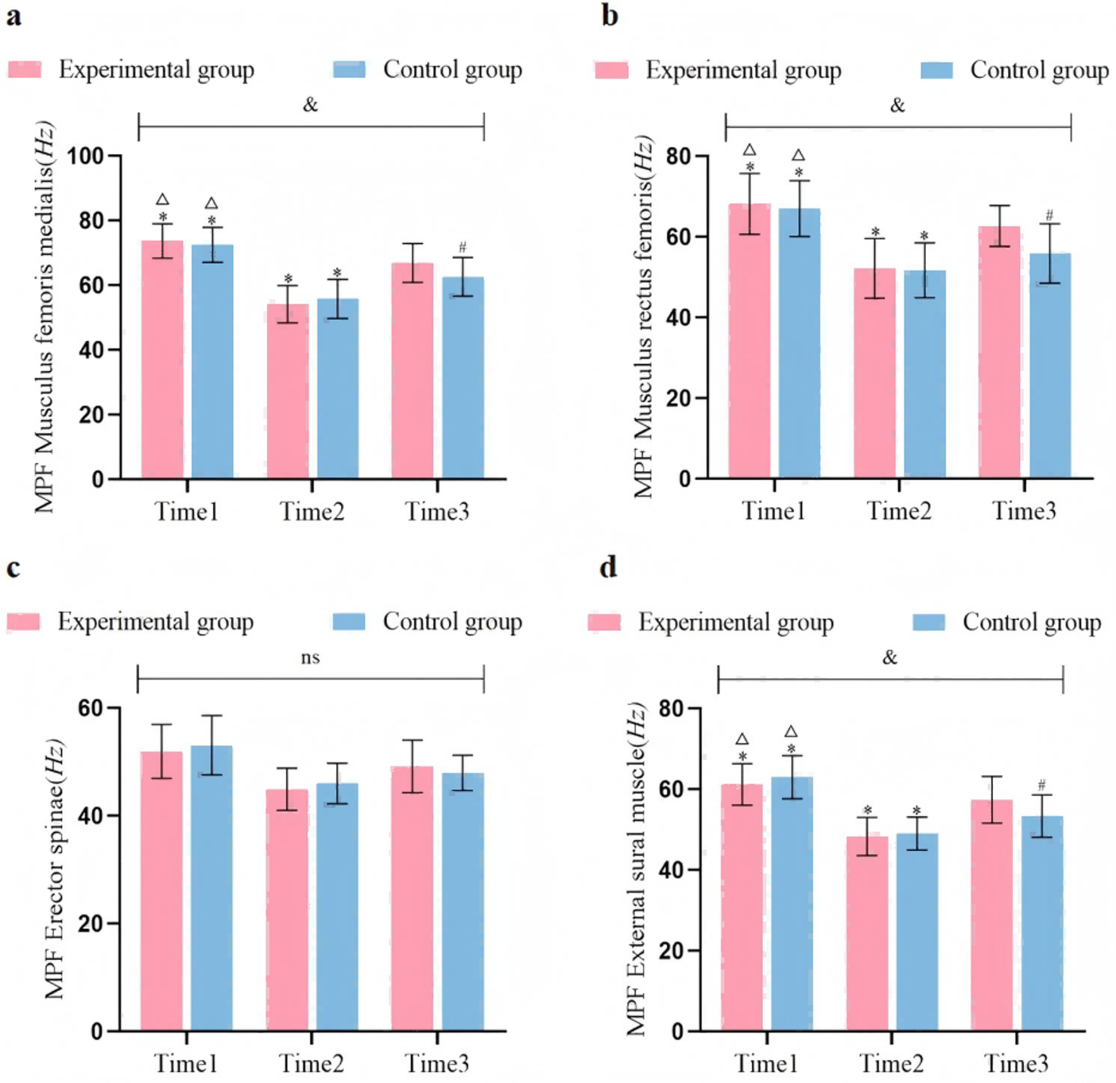
Comparison of sEMG signal MPF between the two groups. (A) Comparison of MPF in the m edial femoral muscle of participants in the two groups; (B) comparison of MPF in the musculus rectus femoris of participants in the two groups; (C) comparison of MPF the erector spinae of participants in the two groups; (D) comparison of MPF in the external sural muscle of participants in the two groups. Note: Compared with the same group after intervention, ^∗^ *P* < 0.05; Compared with the same group after exercise, ^Δ^ *P* < 0.05; compared with the control group at the same time point, ^#^*P* < 0.05; the interaction between time and group, ^&^*P* < 0.05.

There was an interaction between group and time in the MPF of the musculus femoris medialis (*F* = 5.452, *P* = 0.007, *η*^2^_*p*_ = 0.146), musculus rectus femoris (*F* = 3.324, *P* = 0.042, *η*^2^_*p*_ = 0.094), and external sural muscle (*F* = 6.152, *P* = 0.005, *η*^2^_*p*_ = 0.161; Table 8, Fig. 8). The main effects of time on MPF changes in the musculus femoris medialis (*F* = 203.696, *P* < 0.05, *η*^2^_*p*_ = 0.864), musculus rectus femoris (*F* = 67.404, *P* < 0.05, *η*^2^_*p*_ = 0.678), and external sural muscle (*F* = 117.486, *P* < 0.05, *η*^2^_*p*_ = 0.786) were significant. The MPF of the musculus femoris medialis (*P* < 0.05, *η*^2^_*p*_ = 0.121), rectus femoral muscle (*P* < 0.05, *η*^2^_*p*_ = 0.235), and sural muscle (*P* < 0.05, *η*^2^_*p*_ = 0.121) of participants in the control group were lower than those of participants in the experimental group. MPF changes reached significance in the musculus femoris medialis in both the experimental group (*P* < 0.05, *η*^2^_*p*_ = 0.946) and the control group (*P* < 0.05, *η*^2^_*p*_ = 0.883), in the rectus femoral in both the experimental group (*P* <  0.05, *η*^2^_*p*_ = 0.618) and the control group (*P* < 0.05, *η*^2^_*p*_ = 0.766), and in the external sural muscle in just the experimental group (*P* < 0.05, *η*^2^_*p*_ = 0.835). In the musculus femoris medialis, musculus rectus femoris, and external sural muscle, the MPF values within the same group were lower after exercise than before exercise (*P* < 0.05) and after intervention (*P* < 0.05), with values after intervention also being lower than those before exercise (*P* < 0.05).

## Discussion

This study investigated the effects of HBOT and natural oxygen recovery on BFR intervention-induced acute exercise fatigue by examining changes in EEG and sEMG signals. Based on observing the changing characteristics of EEG and sEMG signals, together with conventional fatigue indexes, this study confirmed that BFR intervention induces peripheral muscle fatigue and CNS fatigue simultaneously. Both recovery methods facilitated EEG and sEMG signal, but HBOT accelerated recovery and demonstrated superior effectiveness in fatigue elimination. Unlike passive rest, HBOT specifically mitigates exercise-induced hypoxia, enhances metabolic efficiency, and promotes cellular recovery. Therefore, HBOT intervention can produce a specific intervention effect on exercise-induced hypoxia. In addition, the improvement in both EEG and sEMG signals indicates that HBOT can effectively alleviates central and peripheral fatigue. As such HBOT is a superior intervention for exercise-induced fatigue.

### Effects of HBOT on EEG signal-related indexes after low-load exercise

EEG signals directly reflect the core manifestations of central fatigue, such as cognitive resource depletion and reduced processing speed, by monitoring dynamic activity across specific brain regions (Yang et al., 2020). EEG signals can also accurately and specifically reflect the fatigue state of the CNS, providing a direct basis for the diagnosis of central fatigue (Yang et al., 2020). In addition, EEG tracks the progression of psychological fatigue: as psychological fatigue intensifies, neural activity switches from executive and attentional patterns to default mode, leading to a decrease in α wave frequency and an increase in β wave frequency (Trejo et al., 2015).

**Table 6:** Comparison of sEMG signal RMS indexes between the two groups (*uV*).

Location	Experimental group			Control group			*F*	*P*
	Before exercise	After exercise	After intervention	Before exercise	After exercise	After intervention		
Musculus femoris medialis	165.89 ± 9.32^*^^Δ^	205.89 ± 13.87^*^	174.91 ± 6.18	162.87 ± 7.78^*^^Δ^	208.94 ± 14.88^*^	185.87 ± 6.50^#^	4.859	0.011
Musculus rectus femoris	162.36 ± 5.89^*^^Δ^	197.23 ± 13.58^*^	170.99 ± 5.68	161.70 ± 5.09^*^^Δ^	195.63 ± 11.65^*^	181.02 ± 8.09^#^	7.503	0.001
Erector spinae	153.10 ± 7.65	169.26 ± 9.48	159.49 ± 4.87	151.10 ± 7.40	167.54 ± 9.49	162.51 ± 7.00	1.530	0.225
External sural muscle	158.10 ± 5.42^*^^Δ^	188.58 ± 10.73^*^	169.54 ± 8.48	157.89 ± 8.52^*^^Δ^	185.29 ± 8.86^*^	176.61 ± 8.66^#^	6.141	0.006

**Notes.**

Compared with the same group after intervention.

* *P* < 0.05; Compared with the same group after exercise.

Δ *P* < 0.05; Compared with the control group at the same time point.

# *P* < 0.05; The interaction between time and group.

& *P* < 0.05.

The current study found that after BFR intervention, β-wave activity in the central and occipital regions increased significantly, whereas α-wave activity in the central, occipital, and parietal regions significantly decreased. These changes suggest a decline in neuronal function and altered neurotransmitter release (*e.g.*, 5-hydroxytryptamine), which ultimately induce central fatigue (Zając et al., 2015). This mechanism is closely related to mitochondrial dysfunction, impaired neuroactive amino acid uptake, and metabolic disturbances caused by post-exercise cerebral hypoxia (Kumar & Singh, 2022). Following intervention, the experimental group showed significantly weakened β-waves in central and occipital regions and strengthened α-waves in central, occipital, and parietal regions. These changes were more pronounced with HBOT than with natural recovery, indicating that HBOT accelerates the restoration of neural rhythms and enhances signal conduction efficiency. Thus, HBOT promotes the elimination of central fatigue and improves cognitive processing. Overall, HBOT may exert a regulatory effect on brain electrical signal indicators and CNS function.

The hypoxic state induced by BFR-intervention leads to excessive production of ROS, triggers oxidative stress, and damages mitochondrial DNA and membrane structures (Miura et al., 2017). Meanwhile, it reduces the activity of Na^+^-K^+^-ATPase on the cell membrane, inhibits transmembrane ion flow inside and outside neurons (Simão et al., 2011), decreases the excitability of central nerve cells, and weakens the discharge efficiency of cerebral cortical neurons. HBOT may exerts a positive regulatory effect on the rhythm and amplitude of EEG through core mechanisms such as improving oxygen supply and metabolism of brain tissue, and alleviating oxidative stress response (Groborz, Marsalek & Sefc, 2025). HBOT may increase brain tissue oxygen partial pressure, accelerate the recovery of mitochondrial function and ATP synthesis efficiency, and maintain the stability of neuronal ion channels, providing the energy and ionic basis for the generation of normal EEG signals, thereby accelerating the rate of α-wave elevation (Bin-Alamer et al., 2024). Meanwhile, HBOT mitigates oxidative stress by inhibiting the cyclooxygenase COX-2 signaling pathway, reduces intracranial pressure and tissue compression, restores the synergistic conduction of neural networks, and improves the rhythmic synchrony and spatial coordination of EEG signals, thus accelerating the recovery rate of α and β waves (Lindenmann et al., 2022). In addition, HBOT may also promote lactic acid metabolism in brain cells, correct glucose metabolic disorders in brain tissue, and thereby restore the pH value of extracellular fluid (Zhu et al., 2015). Meanwhile, it reduces glutamate accumulation and the concentration of glutamate in the synaptic cleft, correcting neurotransmitter imbalance (Zhu et al., 2015).

**Table 7:** Comparison of changes of sEMG signal MF indexes between the two groups (Hz).

Location	Experimental group			Control group			*F*	*P*
	Before exercise	After exercise	After intervention	Before exercise	After exercise	After intervention		
Musculus femoris medialis	75.34 ± 7.20^*^^Δ^	54.15 ± 6.59^*^	65.55 ± 5.93	76.34 ± 5.51^*^^Δ^	55.10 ± 7.23^*^	61.21 ± 5.94^#^	3.479	0.037
Musculus rectus femoris	73.57 ± 9.89^*^^Δ^	52.69 ± 4.30^*^	65.85 ± 5.23	74.63 ± 10.21^*^^Δ^	54.59 ± 5.00^*^	62.13 ± 5.08^#^	3.749	0.029
Erector spinae	60.89 ± 5.15	51.38 ± 4.40	56.56 ± 5.45	60.04 ± 3.91	50.49 ± 4.26	54.73 ± 5.45	0.714	0.487
External sural muscle	65.05 ± 5.97^*^^Δ^	53.82 ± 5.29^*^	59.97 ± 6.30	63.46 ± 6.50^*^^Δ^	52.41 ± 4.65^*^	55.61 ± 5.39^#^	3.343	0.045

**Notes.**

Compared with the same group after intervention.

* *P* < 0.05; Compared with the same group after exercise.

Δ *P* < 0.05; Compared with the control group at the same time point.

# *P* < 0.05; The interaction between time and group.

& *P* < 0.05.

### Analysis of the intervention effect of HBOT on sEMG signal-related indexes after low-load exercise

sEMG provides an objective bioelectric assessment of muscle functional state by detecting the summation of skeletal muscle motor unit potentials. It is indicator of peripheral skeletal muscle fatigue. exercise progresses, the proportion of slow muscle fibers involved increases, and their action potential frequency is relatively lower, causing the overall electromyographic signal frequency distribution to shift toward the low-frequency direction, resulting in decreased MPF and MF (Peñailillo, Silvestre & Nosaka, 2013).

**Table 8:** Comparison of sEMG signal MPF between the two groups (Hz).

Location	Experimental group			Control group			*F*	*P*
	Before exercise	After exercise	After intervention	Before exercise	After exercise	After intervention		
Musculus femoris medialis	73.68 ± 5.27^*^^Δ^	54.12 ± 5.75^*^	66.89 ± 6.00	72.43 ± 5.43^*^^Δ^	55.75 ± 6.05^*^	62.59 ± 5.94^#^	5.452	0.007
Musculus rectus femoris	68.16 ± 7.55^*^^Δ^	52.16 ± 7.40^*^	62.68 ± 5.08	66.99 ± 6.90^*^^Δ^	51.71 ± 6.82^*^	55.89 ± 7.37^#^	3.324	0.042
Erector spinae	51.94 ± 5.00	44.91 ± 3.90	49.14 ± 4.87	53.07 ± 5.50	45.96 ± 3.74	47.94 ± 3.28	1.269	0.288
External sural muscle	61.15 ± 5.15^*^^Δ^	48.28 ± 4.74^*^	57.35 ± 5.81	62.95 ± 5.32^*^^Δ^	48.99 ± 4.10^*^	53.36 ± 5.23^#^	6.152	0.005

**Notes.**

Compared with the same group after intervention.

* *P* < 0.05; Compared with the same group after exercise.

Δ *P* < 0.05; Compared with the control group at the same time point

# *P* < 0.05; The interaction between time and group

& *P* < 0.05.

This study found that BFR significantly increased the RMS of the vastus medialis, vastus lateralis, and lateral gastrocnemius; meanwhile, MF and MPF significantly decreased. These changes suggest a decrease in motor unit discharge frequency and a decline in contractile function, characteristic of peripheral fatigue (Farina, Merletti & Enoka, 2014). After peripheral skeletal muscle fatigue, the two groups received different recovery intervention methods. After intervention, the experimental group showed a significant decrease in RMS and a significant increase in MF and MPF. These changes were more pronounced with HBOT than with natural recovery, suggesting that HBOT effectively regulates the electrophysiological state of muscles after exercise and accelerates recovery (Shimoda et al., 2015; Cyr-Kirk & Billaut, 2022).

The hypoxic state induced by BFR intervention not only inhibits the activity of Na^+^-K^+^-ATPase on the muscle cell membrane, but also impairs the function of sarcoplasmic reticulum Ca^2^^+^-ATPase, reducing Ca^2^^+^ processing capacity and causing abnormal cytoplasmic Ca^2^^+^ accumulation (Forouzandehmehr et al., 2024). This disrupts the excitation-contraction coupling process of muscle cells, leading to a leftward shift in the power spectrum of sEMG signals, accelerating the rising rate of RMS and the declining rates of MF and MPF. Hyperbaric oxygen intervention enhances oxygen supply from blood to muscle cells, increases mitochondrial cytochrome c oxidase activity, facilitates efficient operation of the electron transport chain, and improves ATP synthesis efficiency. It accelerates the removal of metabolic wastes such as lactic acid and hydrogen ions, restores the stability of the extracellular fluid environment of muscle cells, and provides a foundation for the generation of normal membrane potentials in muscle cells (Schottlender, Gottfried & Ashery, 2021). Consequently, it delays the downward shift of the sEMG power spectrum, maintains the spectral stability of myoelectric signals, and accelerates the upward recovery rates of MF and MPF. Furthermore, HBOT may also repair damaged motor neurons and optimize the efficiency of central motor command issuance by improving oxygen supply to brain and spinal cord tissues. This enables muscles to recruit fewer and more efficient motor units at the same contractile force, enhancing neuromuscular coupling efficiency, thereby reducing sEMG signal amplitude and accelerating the rate of RMS decline (Bin-Alamer et al., 2024).

This study has several limitations. First, the findings are specific to the five cm cuff width used during BFR; different cuff dimensions may lead to variations in research results. Currently, a cuff width of five cm is commonly used for lower extremity blood flow restriction in relevant studies. A cuff of this width can induce significant ischemia and hypoxia stimulation in the lower limb muscles, while remaining within the tolerable range for the human body. With an increase in cuff width, under the same applied pressure, a wider cuff exerts a significantly stronger restrictive effect on limb blood flow than a narrow one. It may trigger more pronounced cardiovascular responses and vascular function changes, as well as increase participants’ ratings of perceived exertion and pain scores (Rossow et al., 2012), leading to a deeper level of fatigue. Under such conditions, there is insufficient research evidence to support whether hyperbaric oxygen intervention can also effectively promote the recovery of electroencephalographic and electromyographic signals. Therefore, the findings of the present study are only applicable to the alleviation of fatigue induced by a 5-cm cuff width. Second, this study did not perform post-intervention testing, leaving it unclear whether our research results can further improve performance. Subsequent studies should evaluate HBOT recovery across varying cuff widths and further clarify the impact of HBOT recovery on functional performance.

## Conclusion

EEG and sEMG signals indicate that low-load BFR intervention can effectively induce both central and peripheral fatigue. Exposure to 1.3 ATA–HBOT for 60 min promotes recovery of EEG and sEMG signal indices after low-load BFR intervention, accelerating the elimination of central and peripheral fatigue.

##  Supplemental Information

10.7717/peerj.21554/supp-1 Supplemental Information 1 Raw data

## Additional Information and Declarations

### Data Availability

The following information was supplied regarding data availability: The raw data is available in the Supplementary File.
